# Repeatability of small lung nodule measurement in low-dose lung screening: a phantom study

**DOI:** 10.1186/s12880-020-00510-2

**Published:** 2020-10-02

**Authors:** Yu Du, Gao-Feng Shi, Ya-Ning Wang, Qi Wang, Hui Feng

**Affiliations:** Department of Radiology, the Fourth Hospital of Hebei Medical University, No. 12 Jiankang Rd. Changan District, Shijiazhuang, 050011 China

**Keywords:** Computer tomography, Low dose, Pulmonary nodules, Body model, Lung cancer

## Abstract

**Background:**

Lung cancer screening revealed that people with small pulmonary nodules are mostly asymptomatic and that some of these people are at risk of developing lung cancer, so we intended to explore the repeatability of small lung nodule measurement in low-dose lung screening.

**Methods:**

We scanned eight ground-glass nodules (GGNs) and solid nodules, with diameters of 3, 5, 8, and 10 mm. They were divided according to the different combination schemes of tube voltage (KV) and tube current (mA) as 70, 80, 100, and 120 KV, and currents of nine tubes were divided as 20, 30, 40, 50, 60, 70, 80, 90, and 100 mAs.

**Results:**

Compared with the conventional dose group (120 kVp, 100 mAs), the nodule diameter and solid nodule volume measured by all scanning combinations were more consistent (*P* > 0.05), the volumes of 10 mm GGNs combinations were consistent (*P* > 0.05), the volumes of 8 mm GGNs were consistent (*P* > 0.05), the volumes of 5 mm GGNs combinations were consistent (*P* > 0.05), and the volumes of 3 mm were consistent (*P* > 0.05).

**Conclusion:**

In lung cancer screening, CT parameters should be as follows: tube voltage is more than 80 kVp, and tube current is 80 mAs in order to meet the requirements for the accurate measurement of the diameter and volume of pulmonary nodules.

## Background

With the increasing popularity of lung cancer screening, the detection rate of small pulmonary nodules has increased. Lung cancer screening revealed that people with small pulmonary nodules are mostly asymptomatic and that some of these people are at risk of developing lung cancer [1]. Since the detection rate of small pulmonary nodules has increased, the subsequent problem is to determine how to deal with this as early as possible, and additional examination and treatment measures should be avoided. For malignant nodules, early diagnosis can provide a safer and clearer treatment plan. Considering the possibility of false positives, the computed tomography (CT) follow-up and monitoring of small nodules is very important. In addition, the possible radiation risk and economic cost of follow-up should also be comprehensively considered [2]. According to the International Lung Nodule Screening Guidelines, the size and growth rate of nodules are still well-recognized as important indicators to distinguish benign and malignant nodules [3, 4]. Compared with the nodule size in the first examination, the growth rate of the nodule can be calculated, in order to determine its benign and malignant nature. At present, the measurement of nodule size mainly includes diameter measurement, and the latest guidelines take volume measurement as a measurement standard [4, 5].

In the lung cancer screening guidelines, such as the Lung Reporting and Data System (Lung-RADS) and the National Comprehensive Cancer Network (NCCN) guidelines, the mean diameter is used as the size standard for nodal follow-up and treatment [6, 7]. In the Dutch-Belgian Nelson test, volumes serve as a similar standard [5]. Indeed, the determination of the nodule follow-up and treatment plan during the lung cancer screening is not based on the actual size of the nodule in the surgical specimen, but on the size measured on the CT image and the changes before and after the follow-up [8]. Therefore, the present study focuses on the measurement accuracy of low-dose CT for small nodules and determines how to consistently measure the average diameter and volume, in order to determine its size and change, rather than the measurement accuracy of the actual nodule size.

The national lung screening test (NLST) revealed that the use of low-dose CT screening in high-risk groups could reduce lung cancer mortality [9, 10]. According to the size and changes of nodules, lung cancer screening guidelines provide different treatment options. Therefore, in addition to the detection of pulmonary nodules, the repeatability of pulmonary nodule measurement is also an important factor in the follow-up and risk assessment of pulmonary nodules in CT screening. Since annual CT screening increases the risk of radiation-related cancer, the principle of minimizing the CT screening dose is also important [11]. Therefore, it is important to keep the accuracy of the screening image while reducing the radiation dose and avoiding large errors in the detection or measurement of pulmonary nodules.

In lung nodule screening, when using 120 kVp of tube voltage, the tube current can be reduced to less than 100 mAs, on the premise that the image quality can meet the diagnostic requirements. In some studies, the tube current was reduced to 80, 70, 60, or even 10 mAs, and the radiation dose was reduced by 50–84% [12, 13]. Some studies have also reduced the tube current threshold to 20 mAs for pulmonary nodules, including ground-glass nodules screening [14]. Another approach to reducing the radiation dose is to reduce the tube voltage. At present, the most common tube voltage is 100–140 kVp. However, some studies have considered that 80 kVp is feasible for lung nodule screening [15]. Furthermore, few studies have concurrently reduced the tube current and tube voltage in carrying out the lung nodule screening. The phantom experiment is a very helpful method to avoid the extra radiation on patients. Therefore, the present study aimed to investigate the effect of different tube current and voltage combinations in low-dose scanning on the consistency of measurement of the pulmonary small nodule size using phantom. The conventional scanning dose (120 kVp, 100 mAs) was used as the control group.

## Methods

### Chest phantom

The chest model used in the present study (Lungman, Kyoto Kagaku, Tokyo, Japan) was a model that could accurately simulate the human anatomy. The model has a size of 43 × 40 × 48 cm and was designed based on an adult male with a weight of 70 kg. The body model was a male torso model with an artificial mediastinum and trachea, including the pulmonary vessels (right and left) and upper abdomen (diaphragm). The thickness of the chest wall was determined according to the clinical data. The X-ray absorptivity of the substitute material for simulating human soft tissue (polyurethane) and the simulated bone (epoxy resin) were both similar to that of the human tissue. The upper arm was in an abduction position to ensure that the trunk position is suitable for the CT examination. The use of this model can track the direction of the pulmonary vessels in space.

### Simulated pulmonary nodules

For the simulated pulmonary nodules used in the present study, the solid nodules (S, + 100 HU) were made of polyurethane resin, and non-solid nodules (NS, − 800 HU) were made of polyurethane foam resin. In the present study, eight spherical simulated nodules with a smooth surface were used. The diameters were 3, 5, 8, and 10 mm, respectively, the volumes were 14.10, 65.00, 268.00, and 523.00 mm^3^, respectively, and the CT attenuation values were 100 HU and − 800 HU (tube voltage: 120 kVp).

### Image acquisition

A GE Revolution CT scanner [General Electric Co. (GE), USA] was used, and the combined scanning schemes of different tube voltages (kV) and tube currents (MA) were adopted for the phantom. Combinations of tube voltage and tube currents were used. Four tube voltages (70, 80, 100, and 120 KV, respectively) and nine tube currents (40, 60, 80, 100, 120, 140, 160, 180, and 200 mA, respectively) were used. The CT scanning pitch was 0.992:1.000, and the rotation time of the rack was 0.5 s. During the scanning, eight nodules were fixed on the vascular bundle in the phantom with double-sided adhesive tapes. The placement positions were the upper, middle, and lower lungs. The scans were separately performed, and six nodules could be placed for one scan. Each nodule and site were scanned three times, and these were placed in the left and right lungs, respectively. The scanning scope included the whole model from the thoracic entrance to the costophrenic angle. During the scanning process, it was ensured that the scope of each scan was the same. When collecting the images, the Stand and Bone algorithms were used to carry out the adaptive statistical iterative reconstruction (ASIR), in order to obtain the axial image, in which the ASIR ratio was 40%, and both the slice thickness and interval of the reconstruction was 0.625 mm.

### Measurement methods

**Fig. 1:**
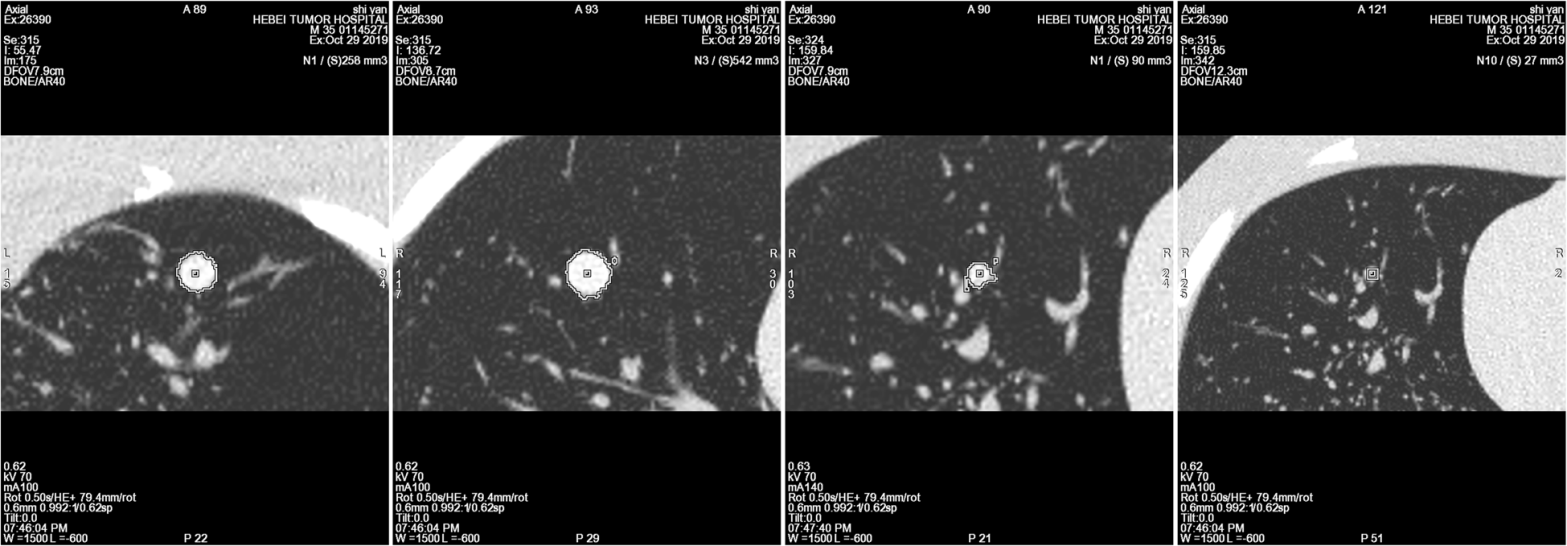
CT images of pulmonary nodules of different sizes

After the end of the scan, all images were imported into the Lung VCAR Single Lesion analysis software AW4.7 workstation (Advantage Workstation, GE, USA), and image processing was performed by a professional imaging physician (8 years of experience in chest imaging diagnosis). The software for pulmonary nodule analysis provided quantitative information on the pulmonary nodule size through volume segmentation for semi-automatic measurement. Apart from clicking again when the software system failed to segment the pulmonary nodules, a manual correction was not performed. The software calculated the diameter (anterior-posterior, left-right, and upper-lower diameters) and the volume of each pulmonary nodule, according to the lesion segmentation (Fig. 1). The average diameter obtained by calculating the average value of three diameter lines has been used in the Lung Cancer Screening Guidelines [16].

### Radiation dose

The radiation dose parameters for the different scanning combinations were recorded: volume CT dose index (CTDI_vol_) and dose length product (DLP). The unit of CTDI_vol_ was mGy. DLP = CTDI_vol_ (mGy) × scan length (cm), and the unit was mGy.cm. An effective dose (ED) meant that the patient received an effective radiation dose during the examination. This was calculated using the following formula: ED = DLP × kn, in which the unit was mSv, and k was the tissue weight factor. According to the European Union’s “CT image quality standard guidelines,” the appropriate weighted tissue factor of a standard chest is 0.017. The CTDI_vol_ and DLP of the different combinations were respectively recorded and calculated, and the ED was calculated.

### Statistics analysis

All experimental data were statistically analyzed using Statistic Package for Social Science 21.0. Measurement data were expressed as mean ± standard deviation (x ± SD). Count data were expressed in percentages (%). The test of normality was conducted using W-tests. The homogeneity of variance was tested using F-tests. The multi-group comparison was conducted using a univariate analysis of variance. The backtesting was conducted using the least significant difference (LSD). Non-normally distributed means of multiple samples or normally distributed means of multiple samples with a heterogeneity of variance were compared using nonparametric tests. Count data were compared using Chi-square tests. *P* < 0.05 was considered statistically significant.

## Results

### Comparison of nodule diameters measured by different scanning combinations

In the present study, a total of 864 scans were completed. The results revealed that compared with the conventional dose group, the measured nodule diameters were in good agreement in all the other groups (all *P* > 0.05, Table 1), and the measured nodule diameters were in good agreement in all the other groups (all *P* > 0.05, Table 2).

**Table 1:** Mean diameter of 10 mm nodules

		Diameter			Diameter		
kV	mAs	10 mm	sd	P	10 mm	sd	P
		GGN			S		
70	20	9.57	0.21	0.898	9.60	0.20	1.000
70	30	10.90	0.61	0.837	9.60	0.20	.830
70	40	12.55	0.64	0.982	9.53	0.21	.056
70	50	9.33	0.15	0.985	10.37	0.86	.079
70	60	9.77	0.31	0.906	10.43	0.80	.830
70	70	10.80	0.44	0.939	9.67	0.12	.747
70	80	10.37	0.68	0.967	9.70	0.10	1.000
70	90	10.00	0.26	0.951	9.60	0.20	.200
70	100	10.20	0.75	0.800	10.00	0.36	.915
80	20	12.20	0.89	0.953	9.63	0.15	.592
80	30	10.70	0.40	0.987	9.77	0.29	.110
80	40	9.73	0.50	0.901	10.10	0.46	.392
80	50	10.87	0.35	0.902	9.87	0.38	.166
80	60	10.67	0.47	0.951	10.03	0.59	.285
80	70	10.20	0.10	0.959	9.93	0.49	.044
80	80	10.10	0.46	0.944	10.23	0.71	.285
80	90	10.30	0.70	0.949	9.93	0.35	.520
80	100	10.23	0.87	0.974	9.80	0.26	.453
100	20	9.90	0.36	0.964	9.83	0.32	.592
100	30	10.03	0.38	0.990	9.77	0.15	.520
100	40	9.70	0.10	0.959	9.80	0.26	.056
100	50	10.10	0.44	0.949	10.20	0.87	.520
100	60	10.23	0.35	0.934	9.80	0.26	.915
100	70	10.43	0.31	0.944	9.63	0.15	.392
100	80	10.30	0.56	0.990	9.87	0.25	.336
100	90	9.70	0.10	0.969	9.90	0.30	.915
100	100	9.97	0.31	0.962	9.63	0.15	.240
120	20	10.07	0.42	0.918	9.97	0.32	.453
120	30	10.63	0.42	0.977	9.83	0.32	.166
120	40	9.87	0.38	0.918	10.03	0.40	.453
120	50	10.63	0.49	0.959	9.83	0.25	.668
120	60	10.10	0.40	0.982	9.73	0.15	.747
120	70	9.80	0.10	0.977	9.70	0.10	.747
120	80	9.87	0.21	0.972	9.70	0.10	.747
120	90	9.93	0.25	0.967	9.70	0.10	.747
120	100	10.00	0.26	–	9.70	0.10	–

**Table 2:** Mean diameter of 8 mm nodules

		Diameter			Diameter		
kV	mAs	8 mm sd	sd	P	8 mm	sd	P
		GGN			S		
70	20	9.10	0.10	.070	8.10	0.70	.068
70	30	8.53	0.91	.068	8.83	0.74	.058
70	40	8.47	0.95	.857	8.00	1.41	.078
70	50	7.97	0.25	.418	8.20	0.56	.058
70	60	8.70	0.75	.753	8.83	0.31	.365
70	70	7.87	0.35	.140	8.50	0.56	.192
70	80	8.20	0.40	.787	8.63	0.72	.420
70	90	7.90	0.30	.892	7.93	0.15	.420
70	100	8.00	0.20	.027	7.93	0.25	.091
80	20	8.77	1.46	.033	8.33	0.12	.840
80	30	8.70	0.10	.964	8.13	0.40	.420
80	40	8.07	0.15	.964	7.93	0.15	.762
80	50	8.07	0.15	.964	8.10	0.40	.687
80	60	8.13	0.21	.097	8.07	0.38	.097
80	70	8.77	0.15	.080	8.90	0.10	.545
80	80	8.73	0.15	1.000	8.00	0.26	.614
80	90	8.10	0.20	.857	8.03	0.31	.481
80	100	7.97	0.12	.210	7.97	0.21	.269
100	20	9.03	0.80	.118	7.83	0.25	.762
100	30	9.07	0.67	.262	8.10	0.20	.365
100	40	8.93	1.36	.323	7.90	0.20	.315
100	50	8.83	0.75	.822	7.87	0.35	.481
100	60	7.93	0.25	.822	7.97	0.12	.365
100	70	7.93	0.15	.964	7.90	0.10	.420
100	80	8.13	0.21	.857	7.93	0.15	.365
100	90	7.97	0.21	.369	7.90	0.26	.687
100	100	8.77	0.74	.822	8.07	0.15	.315
120	20	7.93	0.15	1.000	7.87	0.35	.420
120	30	8.10	0.20	.822	7.93	0.15	.315
120	40	7.93	0.15	.892	7.87	0.21	.481
120	50	8.00	0.26	.928	8.43	0.21	.420
120	60	8.17	0.15	.472	7.93	0.15	.269
120	70	8.63	0.12	.892	7.83	0.06	.420
120	80	8.00	0.10	.822	7.93	0.25	.614
120	90	7.93	0.06	.857	8.03	0.12	.133
120	100	7.97	0.06	–	7.70	0.20	–

Also, compared with the conventional dose group, the measured nodule diameters were in good agreement in all the other groups (all *P* > 0.05, Table 3), and the measured nodule diameters were in good agreement in all the other groups (all *P* > 0.05, Table 4).

**Table 3:** Mean diameter of 5 mm nodules

		Diameter			Diameter		
kV	mAs	5 mm	sd	P	5 mm	sd	P
		GGN			S		
70	20	5.27	0.38	.061	5.07	0.06	1.000
70	30	4.67	0.67	.294	5.07	0.06	1.000
70	40	4.93	0.21	.143	5.07	0.06	1.000
70	50	4.80	0.44	.344	5.07	0.06	.471
70	60	5.57	0.31	.916	5.17	0.12	1.000
70	70	5.23	0.61	.294	5.07	0.06	1.000
70	80	4.93	0.21	.294	5.07	0.06	1.000
70	90	4.93	0.21	.143	5.07	0.06	1.000
70	100	4.80	0.44	.095	5.07	0.06	.151
80	20	4.73	0.35	.143	4.87	0.40	1.000
80	30	4.80	0.44	.294	5.07	0.06	1.000
80	40	4.93	0.21	.294	5.07	0.06	1.000
80	50	4.93	0.21	.294	5.07	0.06	1.000
80	60	4.93	0.21	.673	5.07	0.06	1.000
80	70	5.13	0.67	.294	5.07	0.06	1.000
80	80	4.93	0.21	.294	5.07	0.06	1.000
80	90	4.93	0.21	.294	5.07	0.06	1.000
80	100	4.93	0.21	.143	5.07	0.06	1.000
100	20	4.80	0.44	.294	5.07	0.06	1.000
100	30	4.93	0.21	.143	5.07	0.06	1.000
100	40	4.80	0.44	.117	5.07	0.06	1.000
100	50	5.77	0.76	.248	5.07	0.06	1.000
100	60	5.63	0.51	.143	5.07	0.06	1.000
100	70	4.80	0.44	.294	5.07	0.06	.151
100	80	4.93	0.21	.294	4.87	0.40	1.000
100	90	4.93	0.21	.294	5.07	0.06	1.000
100	100	4.93	0.21	.173	5.07	0.06	1.000
120	20	5.70	0.87	.294	5.07	0.06	1.000
120	30	4.93	0.21	.095	5.07	0.06	.151
120	40	4.73	0.35	.248	4.87	0.40	.000
120	50	4.90	0.20	.344	5.83	0.64	1.000
120	60	4.97	0.15	.143	5.07	0.06	.630
120	70	4.80	0.26	.294	5.00	0.10	1.000
120	80	4.93	0.21	.294	5.07	0.06	1.000
120	90	4.93	0.21	.344	5.07	0.06	.471
120	100	4.97	0.23	–	5.17	0.12	–

**Table 4:** Mean diameter of 3 mm nodules

		Diameter			Diameter		
kV	mAs	3 mm	sd	P	3 mm	sd	P
		GGN			S		
70	20	3.80	0.87	.062	3.23	0.23	.202
70	30	4.53	0.67	.028	2.87	0.21	.202
70	40	4.67	1.12	.034	2.87	0.21	.352
70	50	4.63	0.50	.074	2.97	0.23	.202
70	60	3.10	0.40	.042	3.60	0.26	.352
70	70	3.00	0.44	.074	3.50	0.40	1.000
70	80	3.10	0.40	.089	3.23	0.23	.641
70	90	3.13	0.35	.042	3.10	0.40	.907
70	100	3.00	0.44	.042	3.20	0.44	.641
80	20	3.00	0.44	.391	3.37	0.23	.001
80	30	4.13	0.29	.074	4.20	0.62	.641
80	40	3.10	0.40	.042	3.10	0.40	.641
80	50	3.00	0.44	.042	3.10	0.40	.352
80	60	3.00	0.44	.265	2.97	0.23	.641
80	70	3.37	0.23	.074	3.37	0.23	.641
80	80	3.10	0.40	.042	3.10	0.40	.641
80	90	3.00	0.44	.074	3.10	0.40	.415
80	100	3.10	0.40	.051	3.00	0.44	.641
100	20	3.03	0.50	.089	3.10	0.40	1.000
100	30	3.13	0.35	.023	3.23	0.23	.641
100	40	2.90	0.53	.023	3.10	0.40	.352
100	50	2.90	0.53	.074	2.97	0.23	.641
100	60	3.10	0.40	.074	3.10	0.40	.202
100	70	3.10	0.40	.074	2.87	0.21	.641
100	80	3.10	0.40	.147	3.10	0.40	.202
100	90	3.23	0.23	.147	2.87	0.21	1.000
100	100	3.23	0.23	.147	3.23	0.23	.641
120	20	3.23	0.23	.074	3.10	0.40	.641
120	30	3.10	0.40	.023	3.10	0.40	.641
120	40	2.90	0.53	.074	3.10	0.40	.726
120	50	3.10	0.40	.863	3.33	0.21	.641
120	60	3.73	0.59	.074	3.10	0.40	.815
120	70	3.10	0.40	.074	3.17	0.35	.415
120	80	3.10	0.40	.074	3.00	0.44	.726
120	90	3.10	0.40	.074	3.13	0.35	.641
120	100	3.10	0.40	–	3.10	0.40	–

### Comparison of nodule volumes measured by different scanning combinations

Compared with the conventional dose group, the difference between the combination of 80 kVp and 50 mAs and their combinations were not statistically significant in the 10-mm NS group (*P* > 0.05). The measured nodule volumes were in good agreement between the combination of 70 kVp and 20 mAs, and the above combinations in the 10-mm S group (all *P* > 0.05, Table 5).

**Table 5:** the volume of 10 mm nodules

		Volume			Volume		
kV	mAs	10 mm	sd	P	10 mm	sd	P
		GGN			S		
70	20	293.00	48.75	.000	495.00	13.23	.662
70	30	343.00	37.32	.000	492.33	8.39	.009
70	40	386.33	37.02	.015	511.33	10.02	.000
70	50	371.67	11.06	.840	535.67	26.27	.000
70	60	345.33	6.66	.003	521.33	17.56	.027
70	70	378.33	10.97	.000	508.67	2.89	.001
70	80	387.00	5.57	.000	515.33	6.66	.016
70	90	411.33	6.03	.000	510.00	2.65	.014
70	100	402.00	3.00	.228	510.33	1.53	.585
80	20	357.00	17.06	.000	498.33	3.21	.129
80	30	408.67	4.04	.357	504.33	3.06	.093
80	40	353.67	2.52	.000	505.33	1.53	.093
80	50	521.00	5.29	.000	505.33	2.08	.129
80	60	460.00	5.29	.000	504.33	2.08	.158
80	70	424.67	1.53	.000	503.67	2.08	.074
80	80	434.33	8.02	.000	506.00	6.56	.007
80	90	460.00	7.55	.000	512.00	8.19	.003
80	100	496.00	13.11	.000	513.67	7.64	.093
100	20	393.33	7.09	.000	505.33	5.69	.002
100	30	407.33	15.18	.000	514.33	5.86	.093
100	40	425.67	5.86	.000	505.33	6.11	.001
100	50	437.00	2.00	.000	515.67	5.51	.031
100	60	462.67	6.11	.000	508.33	3.51	.036
100	70	472.00	7.00	.000	508.00	2.65	.007
100	80	488.33	3.21	.000	512.00	5.29	.104
100	90	477.00	3.61	.000	505.00	6.00	.014
100	100	506.67	4.73	.000	510.33	1.53	.007
120	20	416.33	5.13	.000	512.00	3.61	.008
120	30	449.00	17.78	.000	511.67	5.03	.000
120	40	430.33	5.86	.000	517.33	6.11	.007
120	50	463.33	4.51	.000	512.00	4.00	.104
120	60	490.33	4.16	.000	505.00	4.58	.211
120	70	507.33	7.02	.000	502.67	3.21	.009
120	80	499.33	3.51	.000	511.33	2.08	.083
120	90	497.33	3.21	.000	505.67	4.73	.008
120	100	502.33	6.51		511.67	3.21	

Compared with the conventional dose group, the difference between the combination of 80 kVp and 50 mAs and the above combinations was not statistically significant in the 8-mm NS group (*P* > 0.05). The measured nodule volumes were in good agreement between the combination of 70 kVp and 20 mAs, and the above combinations in the 8-mm S group (all *P* > 0.05, Table 6).

**Table 6:** the volume of 8 mm nodules

		Volume			Volume		
kV	mAs	8 mm	sd	P	8 mm	sd	P
		GGN			S		
70	20	183.67	25.42	.001	255.00	1.00	.569
70	30	202.67	9.45	.026	252.33	6.43	.320
70	40	196.67	7.64	.015	250.33	7.02	.137
70	50	198.00	6.56	.000	248.00	13.08	.137
70	60	227.33	15.63	.001	262.00	9.54	.887
70	70	203.67	2.08	.000	255.67	4.04	.393
70	80	239.00	3.00	.000	251.00	7.21	.000
70	90	216.00	3.61	.298	238.00	5.29	.042
70	100	189.67	5.03	.030	245.33	9.50	.434
80	20	196.33	7.09	.004	258.67	4.04	.010
80	30	200.67	3.06	.013	242.67	6.66	.078
80	40	198.33	4.16	.000	246.67	6.81	.007
80	50	209.00	2.00	.000	242.00	2.65	.669
80	60	218.33	3.51	.000	253.00	3.61	.049
80	70	261.33	2.08	.000	264.33	1.53	.943
80	80	268.00	4.00	.000	255.33	5.86	.776
80	90	244.00	7.21	.000	256.33	6.11	.569
80	100	228.00	5.57	.000	257.67	7.51	.067
100	20	217.67	3.06	.000	246.33	4.16	.042
100	30	235.67	5.03	.000	245.33	5.13	.049
100	40	236.33	1.53	.000	245.67	6.66	.057
100	50	261.33	2.08	.000	246.00	7.94	.202
100	60	250.00	12.77	.000	249.00	4.00	.256
100	70	226.67	3.79	.000	249.67	4.16	.355
100	80	235.33	3.21	.000	250.67	1.53	.887
100	90	266.67	3.06	.000	254.33	4.73	1.000
100	100	316.00	4.58	.326	255.00	3.61	.434
120	20	189.33	4.93	.000	251.33	4.93	.943
120	30	237.33	4.73	.000	254.67	4.04	.104
120	40	227.67	8.08	.000	247.33	2.08	.042
120	50	262.67	3.21	.000	264.67	6.66	.202
120	60	255.67	2.89	.000	249.00	2.00	.522
120	70	282.67	4.04	.000	252.00	4.00	.569
120	80	250.33	2.52	.000	252.33	4.16	.887
120	90	255.00	2.00	.000	254.33	4.73	.831
120	100	254.00	5.00	–	254.00	1.73	

Compared with the conventional dose group, the difference between the combination of 80 kVp and 50 mAs and the above combinations was not statistically significant in the 5-mm NS group (*P* > 0.05). The measured nodule volumes were in good agreement between the combination of 70 kVp and 20 mAs, and the above combinations in the 5-mm S group (all *P* > 0.05, Table 7).

**Table 7:** the volume of 5 mm nodules

		Volume			Volume		
kV	mAs	5 mm	sd	P	5 mm	sd	P
		GGN			S		
70	20	33.67	1.53	.709	60.67	4.51	.059
70	30	34.33	3.06	.001	63.67	1.53	.022
70	40	40.00	2.00	.576	64.33	1.53	.396
70	50	34.67	2.52	.000	62.00	2.00	.204
70	60	43.33	2.52	.000	62.67	2.08	.036
70	70	43.67	1.53	.000	64.00	2.00	.004
70	80	44.00	1.00	.000	65.33	1.53	.013
70	90	43.67	2.08	.000	64.67	1.53	.036
70	100	44.33	2.08	.004	64.00	2.00	.671
80	20	39.00	3.61	.138	60.00	1.00	.204
80	30	36.33	1.53	.028	62.67	2.08	.139
80	40	37.67	1.53	.000	63.00	1.00	.092
80	50	42.00	1.00	.000	63.33	1.53	.036
80	60	45.00	1.00	.000	64.00	1.00	.059
80	70	53.00	1.00	.000	63.67	1.53	.000
80	80	49.00	6.56	.000	66.67	1.53	.000
80	90	47.33	1.53	.000	67.00	2.65	.000
80	100	48.67	2.08	.000	69.00	3.61	.013
100	20	43.67	1.53	.000	64.67	1.53	.001
100	30	44.33	1.53	.000	66.33	2.08	.000
100	40	46.33	1.53	.000	66.67	2.08	.036
100	50	49.00	3.61	.000	64.00	3.61	.004
100	60	45.67	3.51	.000	65.33	2.08	.000
100	70	46.67	1.53	.000	67.33	2.08	.092
100	80	48.00	1.00	.000	63.33	1.53	.000
100	90	56.33	1.53	.000	66.67	1.53	.000
100	100	57.67	0.58	.000	66.67	0.58	.002
120	20	51.67	0.58	.000	65.67	1.15	.002
120	30	44.67	0.58	.000	65.67	1.53	.524
120	40	45.33	1.15	.000	61.67	1.15	.289
120	50	55.67	2.08	.000	62.33	0.58	.000
120	60	56.33	2.52	.000	67.33	0.58	.001
120	70	56.33	0.58	.000	66.00	1.00	.000
120	80	57.00	1.73	.000	67.67	0.58	.000
120	90	56.33	2.08	.000	66.67	1.53	.001
120	100	55.67	1.15		66.33	2.08	

Compared with the conventional dose group, the measured nodule volumes were in good agreement between the combination of 80 kVp and 80 mAs, and the above combinations in the 10-mm S group (all *P* > 0.05). The measured nodule volumes were in good agreement between the combination of 70 kVp and 20 mAs, and the above combinations in the 3-mm S group (all *P* > 0.05, Table 8).

**Table 8:** the volume of 3 mm nodules

		Volume			Volume		
kV	mAs	5 mm	sd	P	5 mm	sd	P
		GGN			S		
70	20	16.00	1.00	.003	15.00	1.00	.013
70	30	18.33	0.58	.191	12.00	3.00	.003
70	40	17.00	1.00	.083	11.33	2.52	.003
70	50	17.33	1.53	.000	11.33	1.53	1.000
70	60	15.67	1.15	.000	15.00	1.00	.398
70	70	8.67	0.58	.000	14.00	2.00	.161
70	80	8.67	1.53	.000	13.33	1.53	.398
70	90	8.57	1.15	.000	16.00	1.00	.161
70	100	8.20	1.00	.000	13.33	1.53	.261
80	20	6.33	1.15	.031	13.67	1.53	.000
80	30	15.33	1.15	.000	12.33	1.53	.051
80	40	15.13	0.58	.000	12.67	1.53	.013
80	50	8.67	0.58	.000	12.00	1.00	.003
80	60	8.47	1.53	.000	11.33	1.15	.398
80	70	8.33	1.15	.000	14.00	1.00	.051
80	80	9.00	1.00	.000	12.67	2.08	.093
80	90	10.00	0.00	.000	13.00	2.00	.261
80	100	9.67	1.53	.000	13.67	2.52	.001
100	20	9.00	1.00	.000	11.00	1.00	.013
100	30	9.67	0.58	.000	12.00	1.00	.000
100	40	9.33	0.58	.000	10.67	0.58	.000
100	50	9.67	1.53	.000	10.67	1.53	.000
100	60	10.33	1.15	.000	10.33	1.53	.000
100	70	9.33	0.58	.000	11.33	0.58	.006
100	80	10.67	0.58	.000	11.67	0.58	.000
100	90	11.67	0.58	.000	10.33	0.58	.161
100	100	12.33	0.58	.000	13.33	0.58	.000
120	20	10.33	0.58	.000	10.67	0.58	.001
120	30	9.67	0.58	.000	11.00	1.00	.000
120	40	9.67	0.58	.000	10.00	1.00	.051
120	50	11.67	0.58	.000	12.67	0.58	.006
120	60	12.33	0.58	.000	11.67	0.58	.026
120	70	11.33	0.58	.000	12.33	0.58	.006
120	80	12.67	0.58	.000	11.67	1.53	.026
120	90	12.33	0.58	.000	12.33	1.53	.093
120	100	12.67	0.58		13.00	2.00	

## Discussion

The results of the present study revealed that compared with the conventional dose group (120 kVp and 100 mAs), the measured nodule diameters were in good agreement in all scanning combination groups, but the differences were not all statistically significant. The measured nodule volumes were in good agreement between all scanning combination groups and the conventional dose group, but the differences were not all statistically significant.

Different scanning doses can be obtained by changing the combination of tube voltage and tube current. In the present study, the lowest scanning dose (70 kVp and 20 mAs) was 0.17 mSv, which was only 3.98% of the conventional dose (120 kVp and 100 mAs; 4.24 mSv). For solid and ground-glass small nodules, the difference in the mean diameter of nodules measured by various scanning doses was not statistically significant, the measurement result of the lower scanning dose was in good agreement with that of the conventional dose, and the results revealed that the decrease in scanning dose in a certain range has little impact on the measurement of the mean diameter of nodules.

Compared with the measurement of the nodule diameter, changes in nodule volumes measured by different scanning combinations were relatively complex. For solid nodules with different diameters, even with a lower scanning dose, the results were consistent. For 10-mm ground-glass nodules, better consistency could be obtained by using the scanning combination of more than 80 kVp and 50 mAs. For 8-mm and 5-mm ground-glass nodules, better consistency could be obtained by using the scanning combination of more than 80 kVp and 70 mAs. For 3-mm ground-glass nodules, better consistency could be obtained by using the scanning combination of more than 80 kVp and 80 mAs. With the decrease in scanning dose, the signal-to-noise ratio (SNR) also decreased. In the present study, the segmentation and volume measurement of ground-glass nodules using the pulmonary nodule analysis software was significantly affected, with a decrease in nodule diameter, and this effect was more obvious. Therefore, better consistency could only be obtained by using the scanning combinations of higher tube voltage and tube current. The reason may be because as the tube voltage and tube current decreased, the software had more difficulty accurately segmenting the boundary of the ground-glass nodules. In particular, this was difficult to distinguish from the surrounding vascular structure, resulting in significant differences in volume measurement results. The scanning dose of the combination of 100 kVp and 20 mAs was 0.53 mSv, while the scanning dose of the combination of 80 kVp and 40 mAs was 0.54 mSv. The scanning doses of these two combinations were similar. However, the consistency of the measurement results of the latter to the ground-glass nodule volume was poor. This suggests that compared with the reduction in tube current, the effect of reducing the tube voltage on the measurement of the volume of ground-glass nodules may be greater.

The present study has the following limitations. First, in the present study, the phantom was used for the experiment. Therefore, the conclusion needs to be verified through further clinical applications. The phantom used in the present study was designed based on a 70 kg adult male. Therefore, further studies are needed to determine whether this is suitable for populations with other body types. Second, in the present study, a CT scanner and its supporting software were used to scan and measure the simulated pulmonary nodules. Therefore, further verification is needed to determine whether this is suitable for other types of CT scanners and computer-aided design software. Third, in the present study, the diameters of the simulated pulmonary nodules were 3, 5, 8, and 10 mm, respectively. Although these simulated the solid nodules and ground-glass nodules with the CT attenuation values of 100 HU and − 800 HU (tube voltage: 120 kVp), these could not completely simulate the pulmonary nodules encountered in clinical work, and there were great differences in size, shape, CT attenuation value, and other aspects [6, 17–19]. Therefore, further in-depth studies are needed to verify the conclusions of the present study. Finally, in the present study, the detection rate of small nodules in different combinations of scanning conditions and different doses was not analyzed. Hence, further follow-up studies are needed.

## Conclusion

In lung cancer screening, CT parameters should be as follows: tube voltage is more than 80 kVp, and tube current is 80 mAs, in order to meet the requirements for the accurate measurement of the diameter and volume of pulmonary nodules.

## Data Availability

The datasets used and/or analysed during the current study available from the corresponding author on reasonable request.

## Abbreviations

CT: The computed tomography
Lung-RADS: The lung Reporting and Data System
NCCN: The National Comprehensive Cancer Network
NLST: The national lung screening test
ASIR: The adaptive statistical iterative reconstruction
CTDI_vol_: CT dose index
DLP: Dose length product
ED: EFFECTIVE dose
LSD: The least significant difference
SNR: The signal-to-noise ratio
